# Plasmid-based genetic modification of human bone marrow-derived stromal cells: analysis of cell survival and transgene expression after transplantation in rat spinal cord

**DOI:** 10.1186/1472-6750-7-90

**Published:** 2007-12-14

**Authors:** Mark W Ronsyn, Jasmijn Daans, Gie Spaepen, Shyama Chatterjee, Katrien Vermeulen, Patrick D'Haese, Viggo FI Van Tendeloo, Eric Van Marck, Dirk Ysebaert, Zwi N Berneman, Philippe G Jorens, Peter Ponsaerts

**Affiliations:** 1 Division of Clinical Pharmacology, University of Antwerp, Antwerp, Belgium; 2 Laboratory of Experimental Hematology, Vaccine and Infectious Disease Institute (VIDI), University of Antwerp, Antwerp, Belgium; 3 Laboratory of Physiopathology, University of Antwerp, Antwerp, Belgium; 4 Laboratory of Pathology, University of Antwerp, Antwerp, Belgium; 5 Laboratory of Experimental Surgery, University of Antwerp, Antwerp, Belgium; 6 Centre for Cell Therapy and Regenerative Medicine, Antwerp University Hospital, Antwerp, Belgium

## Abstract

**Background:**

Bone marrow-derived stromal cells (MSC) are attractive targets for *ex vivo *cell and gene therapy. In this context, we investigated the feasibility of a plasmid-based strategy for genetic modification of human (h)MSC with enhanced green fluorescent protein (EGFP) and neurotrophin (NT)3. Three genetically modified hMSC lines (EGFP, NT3, NT3-EGFP) were established and used to study cell survival and transgene expression following transplantation in rat spinal cord.

**Results:**

First, we demonstrate long-term survival of transplanted hMSC-EGFP cells in rat spinal cord under, but not without, appropriate immune suppression. Next, we examined the stability of EGFP or NT3 transgene expression following transplantation of hMSC-EGFP, hMSC-NT3 and hMSC-NT3-EGFP in rat spinal cord. While *in vivo *EGFP mRNA and protein expression by transplanted hMSC-EGFP cells was readily detectable at different time points post-transplantation, *in vivo *NT3 mRNA expression by hMSC-NT3 cells and *in vivo *EGFP protein expression by hMSC-NT3-EGFP cells was, respectively, undetectable or declined rapidly between day 1 and 7 post-transplantation. Further investigation revealed that the observed *in vivo *decline of EGFP protein expression by hMSC-NT3-EGFP cells: (i) was associated with a decrease in transgenic NT3-EGFP mRNA expression as suggested following laser capture micro-dissection analysis of hMSC-NT3-EGFP cell transplants at day 1 and day 7 post-transplantation, (ii) did not occur when hMSC-NT3-EGFP cells were transplanted subcutaneously, and (iii) was reversed upon re-establishment of hMSC-NT3-EGFP cell cultures at 2 weeks post-transplantation. Finally, because we observed a slowly progressing tumour growth following transplantation of all our hMSC cell transplants, we here demonstrate that omitting immune suppressive therapy is sufficient to prevent further tumour growth and to eradicate malignant xenogeneic cell transplants.

**Conclusion:**

In this study, we demonstrate that genetically modified hMSC lines can survive in healthy rat spinal cord over at least 3 weeks by using adequate immune suppression and can serve as vehicles for transgene expression. However, before genetically modified hMSC can potentially be used in a clinical setting to treat spinal cord injuries, more research on standardisation of hMSC culture and genetic modification needs to be done in order to prevent tumour formation and transgene silencing *in vivo*.

## Background

Despite major progress in pharmacological and surgical approaches, a spinal cord injury still remains a very complex medical and psychological challenge, both for patients and their relatives as well as for involved physicians, with currently no existing curative therapy. Next to primary care using surgical osteosynthesis techniques and administration of methylprednisolone [1], further therapeutic approaches are mainly supportive and are focussed on prevention of secondary complications, like urological problems, decubitus, respiratory tract pathology, etc... However, during the past decade, significant progress has been made in animal models of spinal cord injury [2,3], and more therapeutic strategies are likely to be discovered as the existence of an endogenous neural regenerative mechanism in the central nerve system is now generally accepted [4,5]. In this context, a spinal cord injury should not be seen as a single event, but must be recognized as an evolving process with different stages for which different therapeutic approaches can be developed [6]. In general, functional outcome following spinal cord injury will highly depend on the severity of both primary anatomical disruption of nerve tracts (due to contusion, laceration, penetration, etc.) and secondary damage [7] caused by inevitable inflammatory reactions following the initial trauma. In brief, these secondary inflammatory responses mainly consist of an influx of peripheral inflammatory cells (macrophages, T-cells) and an activation of resident microglia. This inflammatory reaction will finally result in the formation of a central cavitation at the site of the initial trauma in the spinal cord surrounded by glial scar tissue. The latter is an important physical and chemical barrier for endogenous regeneration of ascending and descending nerve tracts and thereby compromises functional outcome. The development of future curative treatments will therefore need to combine multiple approaches that are able to modulate secondary inflammation and to enhance endogenous regeneration.

Currently, a very promising experimental strategy for promoting neuronal survival and endogenous regeneration in injured spinal cord is local delivery of neurotrophic factors. Several neurotrophic factors, like brain-derived neurotrophic factor (BDNF), glial cell-derived neurotrophic factor (GDNF), neurotrophin (NT)3 and nerve growth factor (NGF), can stimulate neurogenesis *in vitro *and *in vivo *[8], and their importance for the development of the nervous system, for axonal pathfinding and neuronal survival has made them promising targets to augment regeneration in the injured brain and spinal cord [9,10]. Several approaches have been reported to deliver these neurotrophic factors into injured spinal cord: direct injection [11], adenoviral vectors [12], osmotic minipumps [13-15], fibrin glue [16], hydrogels [17] and genetically modified cell transplants [9,18-20]. Safety, efficacy and applicability of these reported methodologies highly differ between the above-referenced and other published reports, implying the need for continuous study, improvement and validation of *in vivo *delivery systems for neurotrophic factors in spinal cord.

In this study, we investigated the feasibility of a plasmid-based strategy for *in vitro *genetic modification of human bone marrow-derived stromal cells (hMSC) with enhanced green fluorescent protein (EGFP) and NT3. Three genetically modified hMSC lines (EGFP, NT3, NT3-EGFP) were established and used to study cell survival and transgene expression following transplantation in rat spinal cord. First, we present a number of optimised and easy to implement techniques for reproducible histological and molecular detection of hMSC cell transplants in rat spinal cord. Using these techniques we demonstrate that genetically modified hMSC lines can survive in healthy rat spinal cord when using adequate immune suppression and can serve as vehicles for *in vivo *transgene expression. However, transgene silencing and tumour formation by hMSC cell transplants *in vivo *are of utmost importance and should therefore be addressed in priority in future research.

## Results

### In vitro characterisation of genetically modified hMSC cell transplants

**Figure 1:**
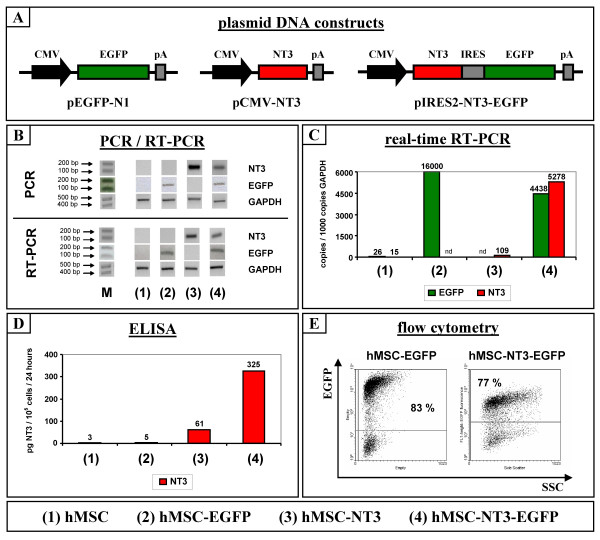
**In vitro characterisation of genetically modified hMSC cell transplants**. (A) Plasmid DNA constructs used for genetic modification of human bone marrow-derived stromal cells (hMSC) in order to obtain hMSC-EGFP, hMSC-NT3 and hMSC-NT3-EGFP cell populations. CMV: Cytomegalovirus immediate early promotor + enhancer. EGFP: enhanced green fluorescent protein. pA: SV40 early mRNA polyadenylation signal. NT3: neurothrophin-3. IRES: internal ribosome entry site. (B) Representative standard PCR and RT-PCR analysis on DNA and mRNA isolated from the different genetically modified hMSC populations used in this study (see numbers below pictures) indicating the presence of transgenic EGFP and/or NT3 DNA and mRNA sequences. M: length marker. GAPDH: glyceraldehyde-3-phosphate dehydrogenase. (C) Representative real-time RT-PCR analysis on mRNA isolated from the different genetically modified hMSC populations used in this study (see numbers below pictures) showing quantitative differences in the level of transgenic EGFP and/or NT3 mRNA transcripts/1000 copies GAPDH; nd: no data available. (D) Representative ELISA measurement on supernatant samples from the different genetically modified hMSC populations used in this study (see numbers below pictures) showing quantitative differences in the level of NT3 secretion in picogram/10^5 ^cells/24 hours. (E) Representative flow cytometric analysis of EGFP expression by hMSC-EGFP and hMSC-NT3-EGFP populations showing quantitative differences in the level of transgenic EGFP protein expression. SSC: side scatter.

A human bone marrow-derived stromal cell line (hMSC) was genetically modified with DNA plasmids encoding either (i) enhanced green fluorescent protein (EGFP) alone, (ii) neurotrophin-3 (NT3) alone, or (iii) both EGFP and NT3, as described in the Materials and Methods section. All three plasmids used for genetic modification were based on the same backbone vector and are shown in Figure 1A. The three genetically modified hMSC lines that were established for use in this study are designated as: hMSC-EGFP, hMSC-NT3 and hMSC-NT3-EGFP. Before transplantation experiments were carried out, transgene expression of these genetically modified hMSC lines was characterised at several passages during culture by PCR, real-time PCR, ELISA and flow cytometry. Figure 1B shows a representative example for the detection of transgenic EGFP and NT3 DNA and mRNA by standard PCR and RT-PCR analysis on DNA and mRNA isolated from the different hMSC lines used in this study. In addition, Figure 1C shows a representative example for the detection of transgenic EGFP and/or NT3 mRNA by real-time RT-PCR analysis on mRNA isolated from all hMSC lines used in this study. With regard to protein expression, Figure 1D shows a representative ELISA measurement of NT3 secretion by the different hMSC lines used in this study and Figure 1E shows a representative flow cytometric analysis of EGFP expression by the EGFP expressing hMSC lines used in this study.

### Immunological survival of hMSC-EGFP cell transplants in rat spinal cord

**Figure 2:**
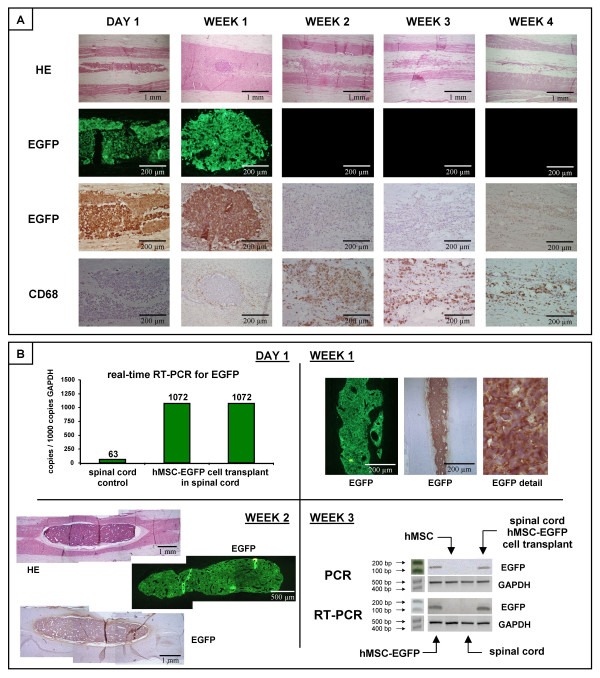
**Immunological survival of hMSC-EGFP cell transplants in rat spinal cord**. (A) Histological assessment of hMSC-EGFP cell transplant survival in rat spinal cord without systemic immune suppression at five time points post transplantation (day 1 and week 1–4). First row: hematoxylin-eosin (HE) staining indicating localisation and general appearance of transplantation site. Second row: direct EGFP fluorescence indicating the presence or absence of EGFP positive hMSC-EGFP cell transplants. Third row: immuno-histochemical staining for EGFP indicating the presence or absence of EGFP positive hMSC-EGFP cell transplants. Fourth row: immuno-histochemical staining for CD68 indicating macrophage infiltration into the transplantation site. All slides were examined using a conventional light/fluorescence microscope and digital pictures were taken under magnification as indicated. Representative pictures were chosen from multiple hMSC-EGFP cell transplanted spinal cords analysed for day 1 (n = 2), week 1 (n = 2), week 2 (n = 2), week 3 (n = 2), and week 4 (n = 6) post-transplantation. (B) Molecular and histological assessment of hMSC-EGFP cell transplant survival in rat spinal cord under systemic immune suppression (subcutaneous 10 mg/kg/day cyclosporin A) at four time points post transplantation (day 1 and week 1–3). DAY 1: Real-time RT-PCR analysis on mRNA isolated from hMSC-EGFP cell transplanted spinal cords on day 1 post-transplantation (n = 2) indicating the presence of EGFP mRNA transcripts *in vivo *in spinal cord following hMSC-EGFP cell transplantation. WEEK 1: direct EGFP fluorescence and immuno-histochemical staining for EGFP indicating the presence of EGFP positive hMSC-EGFP cell transplants on week 1 post-transplantation. Representative pictures were chosen from multiple hMSC-EGFP cell transplanted spinal cords analysed 1 week post-transplantation (n = 5). WEEK 2: HE staining, direct EGFP fluorescence and immuno-histochemical staining for EGFP indicating the presence of EGFP positive hMSC-EGFP cell transplants on week 2 post-transplantation. Representative pictures were chosen from multiple hMSC-EGFP cell transplanted spinal cords analysed 2 weeks post-transplantation (n = 3). WEEK 3: Standard PCR and RT-PCR analysis on DNA and mRNA isolated from hMSC-EGFP cell-transplanted spinal cords 3 weeks post-transplantation indicating the presence of EGFP DNA sequences and EGFP mRNA transcripts *in vivo *in spinal cord following hMSC-EGFP cell transplantation.

Several reports [21-24] ascribe specific immune modulating and immune privileged features to hMSC, suggesting that these cells might serve as a universal off-the-shelf source of cells for use in regenerative medicine and can be transplanted into an allogeneic and xenogeneic host without the need for immune suppressive therapy. In order to investigate whether our established hMSC line has immune modulating properties, two series of transplantation experiments were performed using hMSC genetically modified with the EGFP reporter gene (Figure 1). First, hMSC-EGFP were transplanted in healthy rat spinal cord without systemic immune suppression. At different time points post-transplantation, animals were sacrificed and extracted spinal cords were analysed by histology for cell transplant survival, EGFP transgene expression and macrophage invasion, as presented in Figure 2A. Although on day 1 and week 1 post-transplantation hMSC-EGFP cell transplants were clearly visible by direct EGFP fluorescence and immuno-histochemical staining for EGFP, from 2 weeks post-transplantation the whole transplantation site was invaded with CD68+ macrophages and neither direct EGFP fluorescence nor immuno-histochemical staining for EGFP could indicate the immunological survival of transplanted hMSC-EGFP cells. Interestingly, despite this extensive inflammatory reaction associated with transplant rejection, no negative adverse effects were seen on general health status and locomotion of the cell transplanted animals (data not shown). Next, in order to prevent immunological rejection of hMSC-EGFP cell transplants, the same transplantation experiment was performed but with daily subcutaneous administrations of 10 mg/kg cyclosporine A starting 3 days prior to transplantation. In contrast to the results described above, efficient transplant survival was observed in all cell-transplanted animals at different time points post-transplantation. Representative examples of molecular and histological detection of hMSC-EGFP cell transplants in spinal cord are shown in Figure 2B. Following DNA and mRNA isolation from dissected spinal cord segments, PCR and RT-PCR analysis allowed to detect hMSC-EGFP cell transplants (week 3, PCR for EGFP on isolated DNA) and their transgene expression (week 3, RT-PCR for EGFP on isolated mRNA). In addition, real-time RT-PCR analysis confirmed EGFP transgene expression in spinal cord (day 1, real-time RT-PCR for EGFP on isolated mRNA). Moreover, this molecular observation of hMSC-EGFP cell transplant survival in rat spinal cord and persisting transgene expression at different time points post-transplantation was also proven by histological analysis (week 1 and 2, direct EGFP fluorescence and immuno-histochemical staining for EGFP). In summary, based on the above-described observations, all further transplantation experiments were performed under immune suppression.

### Transgene expression of hMSC-NT3 cell transplants in rat spinal cord

**Figure 3:**
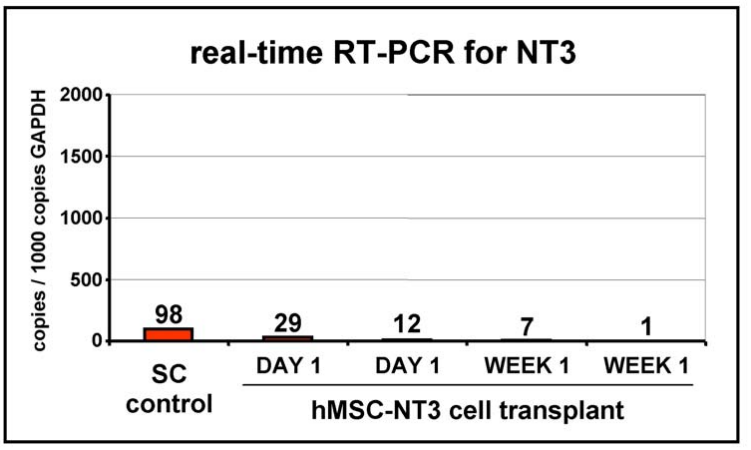
**Transgene expression of hMSC-NT3 cell transplants in rat spinal cord**. Molecular assessment of NT3 mRNA transcription by hMSC-NT3 cell transplants in rat spinal cord. Real-time RT-PCR analysis on mRNA isolated from hMSC-NT3 cell-transplanted spinal cords on day 1 post-transplantation (n = 2) and on week 1 post-transplantation (n = 2) demonstrating the absence of detectable exogenous NT3 mRNA transcripts. SC: spinal cord.

Following genetic modification of hMSC with a DNA plasmid encoding the EGFP reporter gene and successful *in vivo *transplantation and detection of hMSC-EGFP cells, we genetically modified hMSC with a similar DNA plasmid-encoding rat NT3 (see Figure 1A, pCMV-NT3 plasmid). In contrast to the procedures followed for obtaining the presented hMSC-EGFP line (i.e. combined antibiotics selection and cell sorting, see Materials and Methods), obtaining a pure transgene expressing hMSC-NT3 line was not possible through cell sorting, due to the lack of a fluorescent marker molecule (e.g. EGFP). Therefore, following antibiotics selection, single clones were grown and screened by ELISA for highest production of NT3 (Figure 1D, hMSC-NT3 cells). Despite significant production of NT3 by the selected hMSC-NT3 clone, only very low, but however significant, NT3 mRNA was produced by these cells (Figure 1C, hMSC-NT3 cells). However, due to this low level of NT3 mRNA produced by transgenic hMSC-NT3 cells, NT3 transgene expression following transplantation of hMSC-NT3 cells in rat spinal cord could not be detected (Figure 3). In summary, based on the above-described observations, cell sorting for an EGFP reporter gene needs to be included for further genetic modification experiments in order to obtain a transgenic cell population producing high levels of transgenic mRNA.

### Transgene expression of hMSC-NT3-EGFP cell transplants in rat spinal cord

**Figure 4:**
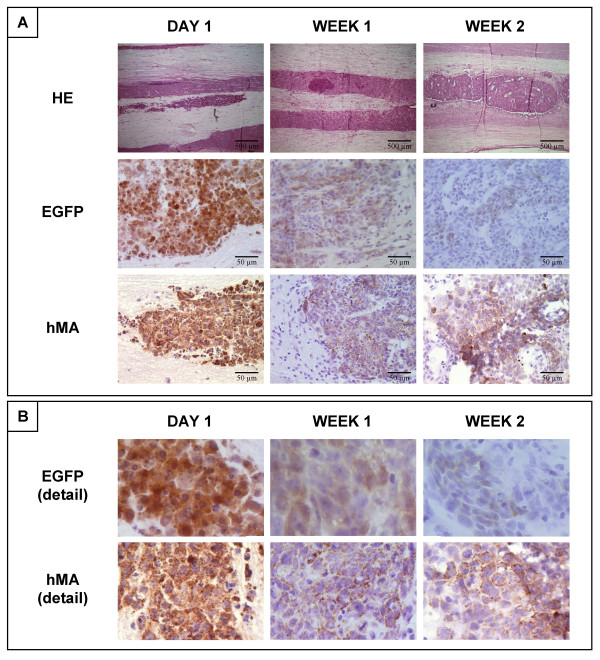
**Transgene expression of hMSC-NT3-EGFP cell transplants in rat spinal cord**. (A) Histological assessment of EGFP expression by hMSC-NT3-EGFP cell transplants in rat spinal cord at three time points post transplantation (day 1, week 1 and week 2). First row: hematoxylin-eosin (HE) staining indicating localisation and general appearance of transplantation site. Second row: immuno-histochemical staining for EGFP showing a decrease in EGFP expression over time by hMSC-NT3-EGFP cell transplants. Third row: immuno-histochemical staining for human mitochondrial antigen (hMA) indicating the human nature of the hMSC-NT3-EGFP cell transplants. Representative pictures were chosen from multiple hMSC-NT3-EGFP cell-transplanted spinal cords analysed 1 day (n = 7), 1 week (n = 11) and 2 weeks (n = 4) post-transplantation. (B) Detail image of the pictures described in part (A) of this figure.

**Figure 5:**
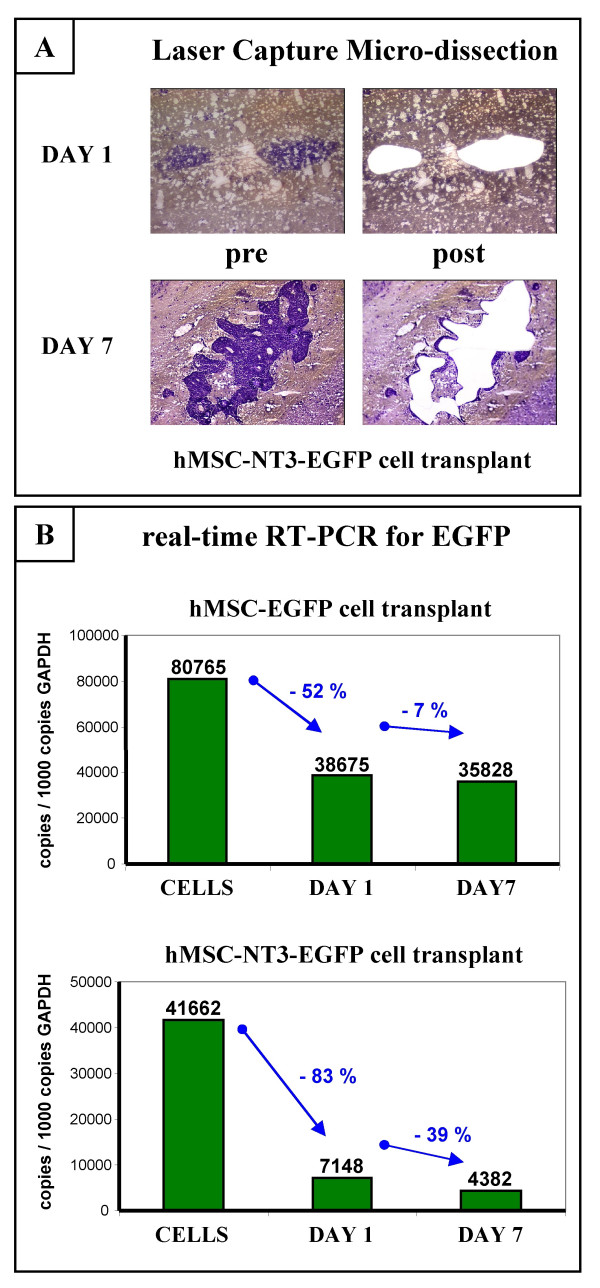
**Analysis of transgene expression by hMSC-NT3-EGFP cell transplants following laser capture micro-dissection (LCM)**. (A) Laser Capture Micro-dissection (LCM) of hMSC-NT3-EGFP cell transplants at day 1 and day 7 post-transplantation in rat spinal cord. Pictures showing cresyl-violet stained slides pre-and post-LCM. (B) Upper graph showing the level of transgenic EGFP mRNA transcripts/1000 copies GAPDH in hMSC-EGFP cell cultures (CELLS, n = 1), on transplanted hMSC-EGFP cells at day 1 post-transplantation (DAY 1, n = 1), and on transplanted hMSC-EGFP cells at day 7 post-transplantation (DAY 7, n = 1). Lower graph showing the level of transgenic EGFP mRNA transcripts/1000 copies GAPDH in hMSC-NT3-EGFP cell cultures (CELLS, n = 1), on transplanted hMSC-NT3-EGFP cells at day 1 post-transplantation (DAY 1, n = 1), and on transplanted hMSC-NT3-EGFP cells at day 7 post-transplantation (DAY 7, n = 1).

In a next attempt to genetically modify hMSC to produce the NT3 neurothrophic factor, we used a DNA plasmid encoding both the NT3 and EGFP protein (Figure 1A, pIRES2-NT3-EGFP plasmid). Because transcription from this plasmid results in the production of one mRNA encoding both proteins, cell sorting for cells expressing high levels of EGFP protein was used in order to obtain an hMSC-NT3-EGFP line producing high levels of EGFP and NT3 mRNA (Figure 1C, hMSC-NT3-EGFP cells) and EGFP and NT3 protein (Figure 1D and 1E, hMSC-NT3-EGFP cells). Following transplantation in rat spinal cord under immune suppression, hMSC-NT3-EGFP cell transplants were easily detectable by morphology on HE-stained slides (Figure 4A). However, while expression of EGFP was clearly demonstrated on day 1 post-transplantation by direct fluorescence microscopy (data not shown) and immuno-histochemical staining for EGFP, EGFP expression on week 1 post-transplantation, as demonstrated by immuno-histochemical staining, was strongly decreased and became almost undetectable by week 2 post-transplantation (Figure 4A and 4B). This decrease in EGFP expression was not due to lack of cell transplant survival because cell transplants could clearly be visualized on HE-stained slides (Figure 4A) and staining with an antibody against human mitochondrial antigen confirmed the human nature of the observed cell transplants (Figure 4A and 4B). In addition, the observed EGFP transgene silencing in hMSC-NT3-EGFP cell transplants, but not in hMSC-EGFP cell transplants, was further demonstrated by laser capture micro-dissection (LCM) experiments. For this, hMSC-EGFP and hMSC-NT3-EGFP cells were transplanted in rat spinal cord under immune suppression. Next, at day 1 and day 7 post-transplantation, transplanted cell populations were isolated by LCM (Figure 5A) and the expression level of EGFP mRNA was analysed by real-time PCR. As suggested by the results presented in Figure 5B, while expression of EGFP mRNA by an hMSC-EGFP cell transplant decreased by 50% at day 1 post-transplantation as compared to cultured hMSC-EGFP cells, EGFP mRNA by an hMSC-NT3-EGFP cell transplant directly decreased by 80% at day 1 post-transplantation as compared to cultured hMSC-NT3-EGFP cells. However, while EGFP mRNA expression then remained stable between day 1 and day 7 post-transplantation for an hMSC-EGFP cell transplant, EGFP mRNA expression further decreased between day 1 and day 7 post-transplantation for an hMSC-NT3-EGFP cell transplant. In summary, based on the above-described results, we observed a similar degree of hMSC-NT3-EGFP cell transplant survival as described above for hMSC-EGFP cell transplants, but transgene expression by hMSC-NT3-EGFP cell transplants rapidly declined *in vivo*.

### Further investigation of transgene silencing in hMSC-NT3-EGFP cell transplants

**Figure 6:**
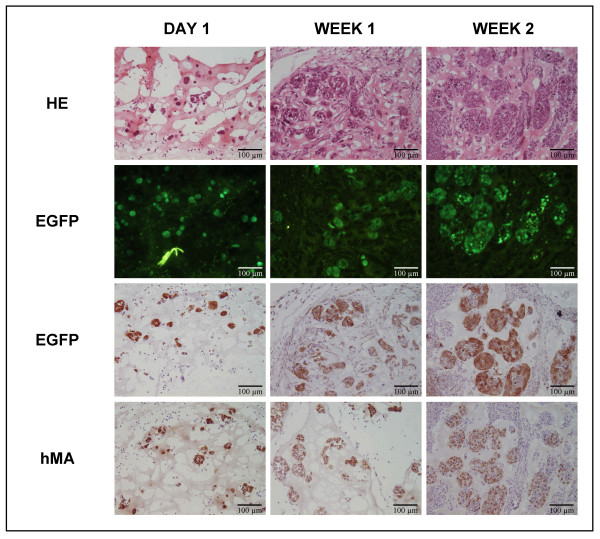
**Transgene expression of subcutaneous hMSC-NT3-EGFP cell transplants**. Histological assessment of EGFP expression by subcutaneous hMSC-NT3-EGFP cell transplants at three time points post transplantation (day 1, week 1 and week 2). First row: hematoxylin-eosin (HE) staining indicating the presence of nucleated cells into the subcutaneously transplanted matrigel. Second row: direct EGFP fluorescence indicating the presence of EGFP positive hMSC-NT3-EGFP cell transplants. Third row: immuno-histochemical staining for EGFP indicating the presence of EGFP positive hMSC-NT3-EGFP cell transplants. Fourth row: immuno-histochemical staining for human mitochondrial antigen (hMA) indicating the human nature of the observed EGFP positive hMSC-NT3-EGFP cell transplants. Representative pictures were chosen from multiple subcutaneous hMSC-NT3-EGFP cell transplants analysed 1 day (n = 6), 1 week (n = 5) and 2 weeks (n = 3) post-transplantation.

**Figure 7:**
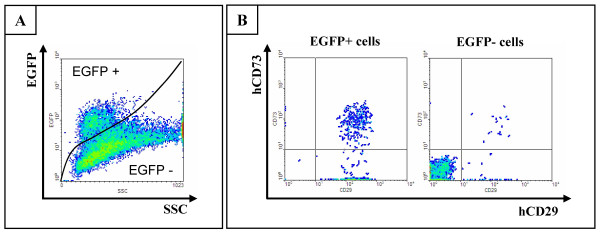
**Transgene expression of re-established hMSC-NT3-EGFP cultures from hMSC-NT3-EGFP cell transplanted spinal cords**. (A) Flow cytometric assessment of EGFP expression (Y-axis) by an established cell culture at passage 1 from dissected hMSC-NT3-EGFP cell transplanted spinal cords showing the presence of both EGFP positive and EGFP negative cells. (B) Flow cytometric staining for human CD29 (X-axis) and human CD73 (= Y-axis) demonstrating that EGFP positive cells originate from hMSC-NT3-EGFP cell transplants while EGFP negative cells do not stain positive for human antibodies. Representative flow cytometric data were chosen from multiple re-established hMSC-NT3-EGFP cell cultures (n = 3).

Since reproducible cell transplantation into spinal cord is a relatively complex procedure, we first investigated whether subcutaneous cell transplantation might provide a valuable alternative in order to study persistence of transgene expression *in vivo*. For this, hMSC-NT3-EGFP cells were transplanted subcutaneously as described in the Materials and Methods section. Unexpectedly and in contrast to the results described above, transgene silencing did not occur in subcutaneous hMSC-NT3-EGFP cell transplants. As demonstrated in Figure 6, no decrease in EGFP expression was observed by direct EGFP fluorescence imaging and immuno-histochemical staining for EGFP. The latter might suggest the presence of certain epigenetic regulatory mechanisms controlling transgene expression *in vivo *in spinal cord. In order to investigate this hypothesis, we assumed that *in vivo *epigenetic silencing of transgene expression is an irreversible process [25]. Thus, in case the observed transgene silencing is due to epigenetic changes (eg DNA methylation and/or histone modifications), a re-established hMSC-NT3-EGFP culture from an EGFP-negative cell transplant should remain EGFP-negative. For this, we re-established hMSC-NT3-EGFP cultures following enzymatic disruption of the cellular context of dissected spinal cords (including site of cell transplantation) at week 2 post-transplantation. When cultures were grown to confluence, the presence and the level of EGFP expression of hMSC-NT3-EGFP was investigated by FACS analysis. Figure 7 shows a representative FACS analysis of an established culture containing both EGFP-positive and negative cells (Figure 7, SSC vs. EGFP dot plot). Further analysis revealed that the EGFP positive cells were indeed hMSC-NT3-EGFP cells as they stained positive for antibodies directed against human CD29 and CD73, both markers present on parental hMSC-NT3-EGFP cells (Figure 7, hCD29 vs. hCD73 dot plot on EGFP-positive cells). EGFP-negative cells present in the cultures displayed a clearly different morphology (data not shown) and presumably were rat fibroblast cells as they were not recognized by antibodies directed against human CD29 and CD73 (Figure 7, hCD29 vs. hCD73 dot plot on EGFP-negative cells). Based on the above-described results, epigenetic modification is most likely not causing the observed transgene silencing in intraspinally transplanted hMSC-NT3-EGFP cells, and a putative mechanism for this observed transgene silencing in hMSC-NT3-EGFP cell transplants in spinal cord (Figure 4), but not subcutaneously (Figure 6), remains unclear.

### Tumorigenicity of hMSC cell transplants in rat spinal cord

**Figure 8:**
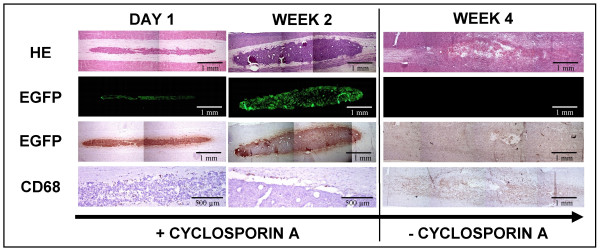
**Tumorigenicity of hMSC-EGFP cell transplants**. Left side: histological assessment of tumour growth by hMSC-EGFP cell transplants in rat spinal cord between day 1 and week 2 post-transplantation in immune suppressed animals (+ cyclosporine A). Right side: histological assessment of tumour regression in rat spinal cord at week 4 post-transplantation upon withdrawal of immune suppression at week 2 post-transplantation (-cyclosporine A). First row: hematoxylin-eosin (HE) staining indicating localisation and general appearance of transplantation site. Second row: direct EGFP fluorescence indicating the presence or absence of EGFP positive hMSC-EGFP cell transplants. Third row: immuno-histochemical staining for EGFP indicating the presence or absence of EGFP positive hMSC-EGFP cell transplants. Fourth row: immuno-histochemical staining for CD68 indicating macrophage infiltration into the transplantation site. Representative pictures were chosen from multiple hMSC-EGFP cell transplanted spinal cords analysed 1 day (n = 4), 2 weeks (n = 3) and 4 weeks (n = 2) post-transplantation.

During progress in this study, we observed tumour growth from on week 1 post-transplantation for both hMSC-EGFP and hMSC-NT3-EGFP cell transplants in spinal cord (see histological analysis on week 2 post-transplantation in Figure 2B, Figure 4 and Figure 8). Because tumour growth is a potential risk in clinical transplantation of autologous, allogeneic and xenogeneic bone marrow-derived stromal cell populations, we investigated whether omitting immune suppressive therapy would be sufficient to prevent further tumour growth and/or destroy malignant cells. As shown in Figure 8, tumorigenic growth of xenogeneic hMSC-EGFP cell transplants at week 2 post-transplantation can efficiently be controlled by the host's immune system. Two weeks following arrest of immune suppressive therapy, the whole transplantation site is invaded by macrophages and no viable cell EGFP+ transplant could be observed.

## Discussion

Stem cells, both embryonic and adult, are attractive targets in the development of cell and gene therapy approaches in a variety of diseases and injuries. While embryonic stem cells have gained much interest due to their pluripotent differentiation capacity, the potential clinical use of multipotent adult stem cell populations in regenerative medicine seems to be more realistic taking into account certain immunological, practical and ethical advantages. Currently, our research is focussing on the use of adult bone marrow derived stromal cells (MSC) as a cellular minipump for neurotrophic factors in injured spinal cord in order to promote neural regeneration. In this context, the aim of this study was to investigate the feasibility of a plasmid-based strategy for genetic modification of human (h)MSC with enhanced green fluorescent protein (EGFP) and/or neurotrophin (NT)3, and to study *in vivo *cell survival and transgene expression of genetically modified hMSC following transplantation in rat spinal cord.

In the first part of this study, we investigated survival of hMSC-EGFP cell transplants in rat spinal cord in immune competent and immune suppressed rats. Survival of hMSC-EGFP in normal spinal cord of immune competent rats was significantly compromised from an early time point (day 1 post-transplantation) and resulted in total graft eradication from on week 2 post-transplantation (Figure 2A). However, suppressing the immune system with cyclosporine A resulted in successful long-term *in vivo *graft survival (Figure 2B). The observed immunological rejection of MSC in the absence of systemic immune suppression is in contrast to a large amount of literature suggesting potential *in vivo *immune modulation and immune privileged properties of MSC [21-24]. However, we believe that there is a common misunderstanding regarding the immune privileged nature of MSC. While these cells have shown immune suppressive effects on Graft-versus-Host disease when given intravenously as shown in several publications, currently, as also demonstrated by the presented results, there is no strong evidence that these cells are of such an immune tolerant nature that they can be transplanted in allogeneic and xenogeneic hosts without giving proper immune suppression. The latter was recently also suggested by Eliopoulos *et al.*[26], who clearly demonstrated that mice implanted with MHC-mismatched MSC-expressing murine erythropoietin, in contrast to mice implanted with syngeneic MSC-expressing erythropoietin, failed in causing a sustained rise in hematocrit level. In addition, Swanger *et al.*[27] demonstrated improved survival of transplanted bone marrow stromal cells in spinal cord when using high-dose cyclosporine A, indicating not only the importance of immune suppression, but also a link between cell survival and level of immune suppression. Therefore, based on these published reports and supported by our findings, we conclude that xenogeneic hMSC cell transplantation in rat is feasible and long-term transplant survival can be achieved under appropriate immune suppression.

In the second part of this study, we investigated the stability of EGFP or NT3 transgene expression by hMSC-EGFP, hMSC-NT3 and hMSC-NT3-EGFP cell transplants in rat spinal cord. While *in vivo *EGFP expression of transplanted hMSC-EGFP cells was readily detectable at different time points post-transplantation (Figure 2B), *in vivo *transgene expression by hMSC-NT3 and hMSC-NT3-EGFP cells was, respectively, undetectable (Figure 3) or declined rapidly between day 1 and 7 post-transplantation (Figure 4). *In vivo *transgene silencing is frequently reported and is currently considered as a major obstacle in the development of *ex vivo *gene therapy protocols using stem cells. One possible explanation for this transgene silencing might be epigenetic modification of introduced transgenic DNA sequences. Transgene silencing can be initiated and sustained by: (i) methylation of CpG-rich DNA sequences which inhibits RNA transcription, or (ii) deacetylation of histones which allows DNA condensation and makes DNA inaccessible for RNA transcription [28]. In our study, we used an internal ribosome entry site (IRES)-based construct for simultaneous expression of EGFP and NT3 protein starting from a single messenger RNA. Others previously reported similar transgene silencing when using IRES-based constructs, however *in vivo *transgene silencing could be reversed when animals were treated with 5-azacytidine, a methyltransferase inhibitor [29]. In addition, also deacetylase inhibitors (e.g. trichostatin A and valproic acid) have demonstrated a capacity to prevent transgene silencing *in vitro *and *in vivo *[30,31]. However, in a first trial experiment, neither *in vitro *treatment of hMSC-NT3-EGFP cells, nor *in vivo *treatment of cell-transplanted rats, with 5-aza-2-deoxycytidine (a less toxic analogue of 5-azacytidine [32]), trichostatin A or valproic acid could reverse the observed transgene silencing *in vivo *in spinal cord (data not shown). Next, further analysis towards a putative explanation for the observed transgene silencing indicated: (i) that transgene silencing did not occur in subcutaneously transplanted hMSC-NT3-EGFP cells (Figure 6), and (ii) that silenced transgene expression was reactivated upon re-establishment of hMSC-NT3-EGFP cultures out of cell-transplanted spinal cords (Figure 7). Based on these observations, we conclude that the observed transgene silencing in our hMSC-NT3-EGFP cell transplants is not due to epigenetic changes, but is most likely due to a natural site-dependent adaptation of transgene expression *in vivo*. Therefore, in order to use MSC or fibroblast cell transplants as cellular minipumps, genetic modification needs to result in sufficiently high transgene expression able to persist even when cellular activity might be reduced in spinal cord [19,33].

In the third part of this study, because we observed a slowly progressing tumour growth following transplantation of all our hMSC cell transplants, we investigated whether omitting immune suppressive therapy is sufficient to prevent further tumour growth and to eradicate malignant xenogeneic cell transplants. Despite some tumorigenic characteristics of our hMSC cell transplants (high degree of Ki67-positivity, multiple mitoses, central necrosis, neovascularisation,... (data not shown)), total regression was seen over a 2-week period after cessation of systemic immune suppression. In addition, despite the extent of the resulting inflammatory infiltration in the spinal cord, we did not observe any influence on functional behaviour of the animals (data not shown). Of note, the observed tumour formation was not totally unexpected given the fact that prolonged *in vitro *stem cell culture might favour growth of MSC clones which potentially can become tumorigenic in vivo as reported by several investigators [34-37]. This *in vitro *culture-based selection of potentially tumorigenic MSC clones might indeed be a serious problem when aiming transplantation of gene-marked autologous or allogeneic MSC populations under immune suppression, where the immune system will not be able to control tumorigenicity. However, this does not mean that all hMSC cultures will behave this way. Additional experiments that were performed with non-modified early passage hMSC cultures indicated excellent cell survival with no signs of tumour formation upon transplantation into healthy spinal cord (data not shown). The absence of in vivo tumour formation for early passage MSC cultures has also been reported by other investigators [34,37]. Therefore, based on these and other published reports/reviews about mesenchymal stromal cells [38] and supported by our findings, we conclude that new techniques need to be developed in order to safely culture and genetically modify hMSC cell transplants for research, and eventually clinical applications.

## Conclusion

In this study, we demonstrate that genetically modified hMSC lines can survive in healthy rat spinal cord over at least 3 weeks by using adequate immune suppression and can serve as vehicles for transgene expression. However, before genetically modified hMSC can potentially be used in a clinical setting to treat spinal cord injury, more research on standardisation of hMSC culture and genetic modification needs to be done in order to prevent tumour formation and transgene silencing *in vivo*.

## Methods

### Human bone marrow-derived stromal cells (hMSC)

Four cryopreserved human bone marrow-derived stromal cell cultures used in a previously published study [39] were thawed and cultured in complete culture medium (CCM) consisting of Iscove's modified Dulbecco's medium supplemented with 2 mM L-glutamine (IMDM; Cambrex), 100 U/ml penicillin (Invitrogen), 100 mg/ml streptomycin (Invitrogen), 1.25 mg/ml amphotericin B (Invitrogen) and 10% fetal calf serum (FCS; Hyclone). In one out of four cultures, outgrowths of one or more cells lead to the establishment of an immortal bone marrow-derived stromal cell line. This line was (i) analysed by immunostaining and found to be CD166+, CD44+, CD29+, CD73+, CD10+, HLA ABC+ and CD34-, CD45-, MHCII-, CD31-, CD13-, and (ii) analysed by *in vitro *adipogenic and osteogenic differentiation studies and found to have lost their *in vitro *differentiation potential. This parental line, designated as "hMSC", was further cultured in CCM in T75 culture flasks (corning) at 37°C in a humidified atmosphere supplemented with 5% CO_2_. For splitting, cells were harvested once a week using Trypsin/EDTA (Invitrogen) treatment and replated at a concentration of 6 × 10^3 ^cells/ml in 20 ml CCM in a new T75 culture flask.

### DNA plasmids

The following DNA plasmids were used in this study for genetic modification of cultured hMSC: (i) the commercially available pEGFP-N1 plasmid encoding the enhanced green fluorescent protein (EGFP) (Clontech), the pCMV-NT3 plasmid encoding the rat neurothrophin-3 (NT3) protein, and (iii) the pIRES2-NT3-EGFP plasmid encoding rat NT3 and EGFP. The pCMV-NT3 plasmid was constructed by replacing the IRES-EGFP sequence from the pIRES2-EGFP plasmid (Clontech) with the rat NT3 cDNA (pAd-EF-NT3 plasmid, kindly provided by Prof. HD Shine, Baylor College of Medicine, Houston, TX, USA) [40]. The pIRES2-NT3-EGFP was constructed by inserting the NT3 cDNA into the multiple cloning site of the pIRES2-EGFP plasmid. After selection of successfully ligated pCMV-NT3 and pIRES2-NT3-EGFP clones via restriction digest mapping, several clones were confirmed by sequence analysis. All plasmids were propagated in *E. coli *supercompetent cells (Stratagene) and purified using plasmid midiprep columns (Qiagen). Directly before use in electroporation experiments, the plasmids were purified again using a PCR purification kit (Qiagen) and resuspended in DNA/RNA-free H_2_O at a concentration of 0.3 μg/μl.

### Stable genetic modification of hMSC

Stable genetic modification of hMSC following plasmid DNA electroporation was performed by culture under combined antibiotics selection, fluorescence activated cell sorting (FACS) and/or single cell cloning [41]. Briefly, hMSC were harvested, washed twice with CCM, and resuspended at 5 × 10^6 ^cells/ml in OptiMem medium (Invitrogen) supplemented with 10% FCS. Next, 500 μl cell suspension was mixed with 10 μg plasmid DNA in a 4 mm electroporation cuvette (Thermo Electron) and electroporation at 260 V and 1050 μF was carried out using a mammalian cell electroporation device (EquiBio). Following electroporation, cells were directly resuspended in CCM and cultured for 48 hours. Next, medium was changed to CCM supplemented with 250 μg/ml neomycin-analogue G418 (Sigma) for 3–4 weeks of selection. Next, in order to establish polyclonal "hMSC-EGFP" and "hMSC-NT3-EGFP" lines, cells highly positive for EGFP (highest 5%) were sorted twice using a FACS-Vantage cell sorter (Becton Dickinson). For establishment of a clonal "hMSC-NT3" line, single clones were grown and screened for NT3 expression by ELISA and RT-PCR (see further). All three lines were further cultured in CCM supplemented with 250 μg/ml G418 in T75 culture flasks at 37°C in a humidified atmosphere supplemented with 5% CO_2_. For splitting, cell were harvested once a week using trypsin/EDTA treatment and replated at a concentration of 6 × 100^3 ^cells/ml in 20 ml CCM in a new T75 culture flask.

### Flow cytometry

Flow cytometric analysis was used for routine (weekly) and pre-transplant measurement of EGFP transgene expression and cell viability of harvested genetically modified hMSC populations. For this, a sample (0.5 × 10^6 ^cells) of harvested cells was resuspended in 1 ml CCM and analysed for EGFP expression on a FACS-scan analytical flow cytometer (Becton Dickinson). Cell viability was measured after addition of 1 μl/ml propidiumiodide (1 mg/ml stock solution, Sigma) to the cell suspension directly before flow cytometric analysis. In some experiments, in order to discriminate hMSC within fibroblast/neural cell cultures derived from dissected spinal cord (see below), cell samples were stained with a phycoerythrin (PE)-labelled monoclonal anti-human CD73 (Becton Dickinson) and a PE-Cy5 labelled monoclonal anti-human CD29 (Becton Dickinson) antibody. For this, cell samples (1 × 10^6 ^cells/staining) were washed twice with Phosphate Buffered Saline (PBS) supplemented with 1% FCS, and resuspended in 100 μl PBS + 1% FCS. Next, 1 μg of each antibody was added for 15 min., followed by a washing step with PBS + 1% FCS. Finally, cells were resuspended in 1 ml PBS + 1% FCS and analysed on a FACS-scan analytical flow cytometer.

### ELISA

Secretion of the NT3 neurotrophic factor by hMSC-NT3 and hMSC-NT3-EGFP cells *in vitro *was determined using the NT3 Emax ImmunoAssay Systems (Promega), according to manufacturers' instructions.

### Preparation of cell transplants

Following harvesting of hMSC-EGFP, hMSC-NT3 and hMSC-NT3-EGFP cell populations via trypsin/EDTA treatment, cells were washed twice with (PBS) supplemented with 5% FCS. Next, cells (mean viability of cell populations: 75–90%) were resuspended in PBS + 5% FCS at a concentration of 100 × 10^6 ^cells/ml for intraspinal cell transplantation. Cell preparations were kept at room temperature until injection. For subcutaneous transplantation, cells were resuspended in a cooled (1–4°C) Matrigel solution (Becton Dickinson, dilution: 1/2 Matrigel + 1/2 PBS+5%FCS) at a concentration of 100 × 10^6 ^cells/ml. Cell preparations were kept on ice until injection.

### Animals

Female Wistar rats (n = 75, Charles River Laboratories), starting weight 175–200 grams, were divided ad random into different experimental groups. For all experiments, rats were kept in normal day-night cycle (12/12) and got food and water ad libitum. All experimental procedures were approved by the "Ethical Committee for Animal Experiments" of the Antwerp University (approval no. 2004/68).

### Intraspinal cell transplantations

All surgical interventions were done under proper sterile conditions. One hour before general induction, rats were premedicated by subcutaneous (SC) injection of buprenorphine (0.1 mg/kg), five minutes before skin incision cefazoline (25 mg/kg) was injected subcutaneously and repeated after 6 hours in order to prevent post-operative infections. Animals were then anaesthetized in an induction chamber with a mixture of O_2 _and N_2_O (0.5 l/min/1.0 l/min) and 4.0% isoflurane (Forene, Abbott). During surgery, anaesthesia was maintained by using the same mixture of O_2 _and N_2_O (0.5 l/min/1.0 l/min) and 0.75% isoflurane (Forene) by a face mask. After skin incision and spreading of the paraspinal muscles a laminectomy was performed on Th10–Th11, using a 10× Zeiss OpMi1 operation microscope. Afterwards an automatic micro-injector pump (kdScientific) with 10 μl Hamilton Microliter™ Syringe was positioned above the exposed dura. A 33-Gauge Hamilton needle, attached to the syringe was stereotactically placed through the intact dura on midline position on a depth of 1.0 mm. After 2 minutes of pressure equilibration 5 μl cell suspension (containing 5 × 10^5 ^cells) was injected over 5 minutes (1 μl/min). Again a waiting period of 2 minutes with the needle still in position in the spinal cord was used for pressure equilibration and prevention of backflow of cell suspension. Muscle and skin was closed with respectively Vicryl (Ethicon) and staplers after proper desinfection of the operation field. Postoperative application of 10 ml glucose 5% solution prevented possible dehydration. During surgery, body temperature was kept on 37% by using a heating path with feedback control by an intrarectal placed sensor. Operated animals recovered by a 5 minutes period of 100% O_2 _and were placed individually in plastic cages till day 1 postoperative with ad libitum water and food. Skin staplers were removed 6 days post-operative. Daily follow-up was done by measuring weight and assessment of general health status. During the entire experiment, immune mediated rejection of cell transplants was prevented by daily subcutaneous injections of 10 mg/kg cyclosporine A.

### Subcutaneous cell transplantations

Subcutaneous injections were done in the interscapular region. After shaving the region of interest, 300 μl of fluid matrigel-cell suspension was injected after which the matrigel immediately coagulated to a palpable subcutaneous tumour. During the entire experiment, immune mediated rejection of cell transplants was prevented by daily subcutaneous injections of 10 mg/kg cyclosporine A.

### Spinal cord dissection for molecular and cellular analysis

At different time points post-transplantation, animals were re-anaesthetised as described above. After exposing the cell injection site, an expansion of the previous laminectomy of Th10 was performed to a total of three thoracal levels (e.g. Th 9–10–11). The spinal cord was cut 5 mm cranial and 5 mm caudal from the injection site. This 10 mm section was then extracted and further processed. (i) For standard and real-time PCR/RT-PCR analysis on whole spinal cord sections, the extracted spinal cord sections were preserved in RNAlater (Ambion), as described by the manufacturers' instructions, until further processing (see below). (ii) For gene expression analysis following Laser Capture Micro-dissection (LCM), extracted spinal cord sections were immediately frozen into liquid nitrogen and stored at -80°C until further processing (see below). (iii) For cellular analysis of transplanted cell populations, extracted spinal cord sections were washed in PBS and incubated for 90 min in a 0.1% collagenase (Sigma) solution dissolved in PBS. Next, dissociated cells were plated in CCM in T75 culture flasks. When cultures reached confluence, human cells were stained with antibodies against human CD73 and CD29 and analysed for EGFP expression using a FACS-scan analytical flow cytometer (see above).

### Spinal cord dissection for histological analysis

Rats were deeply anaesthetized by intraperitoneal injection of sodium pentobarbital (90 mg/kg) and then perfused transcardially with 150 ml heparinised (1 U/ml) NaCl 0.9%, followed by 300 ml of cold buffered paraformaldehyde 4% (pH 7.4) over a period of 25 min. The spinal cord was than excised from low lumbal towards high cervical level, including the injection site, and immersed for an additional 2 hours in the same fixative.

### Matrigel dissection

Subcutaneously placed matrigels were removed under gas anaesthesia and processed for molecular or histological analysis as described above.

### Standard and real-time PCR/RT-PCR analysis on cells and whole spinal cord sections

Genetically modified hMSC cell cultures and dissected spinal cord sections were processed for simultaneous DNA and RNA isolation using an AllPrep DNA/RNA Mini Kit (Qiagen), as described by the manufacturers' instructions. Following spectrophotometric quantification, isolated DNA was directly used for further PCR analysis. Isolated RNA was first reverse-transcribed into cDNA for 1 hour using an Omniscript RT kit (Qiagen), as described by manufacturers' instructions. Next, both DNA and cDNA was analysed for the presence of GAPDH, EGFP and NT3 sequences by standard PCR on a Thermocycler Px2 machine (Thermo Cycler) or by real-time PCR on an iCycler Thermal Cycler machine (Biorad). The following primer pairs were used for specific amplification: for GAPDH forward 5'-ACC ACA GTC CAT GCC ATC AC-3' and reverse 5'-TCC ACC ACC CTG TTG CTG TA-3' (this primer pair recognizes both rat and human GAPDH sequences), for EGFP forward 5'-AGA ACG GCA TCA AGG TGA AC-3' and reverse 5'-TGC TCA GGT AGT GGT TGT CG-3', and for NT3 forward 5'-GAT CCA GGC GGA TAT CTT GA-3' and reverse 5'-AAT CAT CGG CTG GAA TTC TG-3' (combination of this primer pair recognizes rat NT3 sequences, not human NT3 sequences; used for results described in Figure 1B) or reverse 5'-CTT ATC ATC GTC ATC CTT GTA GTC-3' (use of the latter primer recognizes a FLAG sequence on transgenic rat NT3 sequences, not on endogenous rat NT3 sequences; used for results described in Figure 1C and Figure 3). Standard PCR reactions were setup using a Taq PCR Core Kit (Qiagen) and real-time PCR reactions were setup in duplicate using a SYBR Green ER qPCR Supermix (Invitrogen), both according to the manufacturers' instructions. Reactions were carried out as follows: after an initial denaturation step at 94°C for 4 min., amplifications consisted of 30 (standard PCR) or 60 (real-time PCR) cycles of denaturation at 95°C for 1 min., annealing at 56°C for 1 min. and extension at 72°C for 1 min. PCR products obtained after standard PCR were analysed in a 1% agarose gel stained with 0.5 μg/ml ethidiumbromide and visualised by UV light. For analysis of real-time PCR data, expression levels of EGFP and NT3 were calculated versus expression of GAPDH.

### Gene expression analysis following laser capture micro-dissection (LCM)

Frozen spinal cords were embedded in Neg-50™ (Richard-Allan Scientific) and 8 μm thick sections were cut using a cryomicrotome (Microm HM 500). These sections were then caught on a PEN-membrane, stretched on a metal frame, and instantly fixed with a 70% ethanol solution to inhibit RNA degradation. Next, sections were stained with cresyl-violet (LCM Staining Kit, Ambion) and dried in xylene. During the cutting process, some sections were stained with hematoxylin-eosin to verify the presence of the injected cells. The frames were sandwiched with an RNase-free slide and mounted on the LCM microscope (SL μCut, MMI). Areas with cells of interest in a range from 0.5 mm^2 ^to 1.5 mm^2 ^were selected with a software interface at 400× magnification and cut with a UV-laser. RNA was extracted using the Picopure™ RNA Isolation kit (Arcturus), and the RNA quality was assessed using the Agilent 2100 BioAnalyzer (Agilent Technologies). The overall RNA quality was very good and the calculated yields ranged from 3 – 10 ng of total RNA. RNA was reverse transcribed using the Sensiscript RT Kit (Qiagen). Real-Time PCR was carried out in triplicate on an ABI Prism 7700 Sequence Detection System (Applied Biosystems) using a custom Taqman™ Primers-Probes set for the EGFP gene and a Primers-Probe set for GAPDH as housekeeping gene. Real-time PCR amplification was carried out using following scheme: 2 initial steps of 2 min at 50°C and 10 min at 95°C, then 55 amplification loops with a denaturation of 1 sec at 95°C and an annealing and extention phase of 1 min at 60°C in a reaction volume of 25 μL.

### Histological analysis

Fixed spinal cord segments and matrigels were dehydrated in sucrose gradients (5%, 10% and 20%), frozen in liquid nitrogen and stored at -80°C until further processing. Consecutive 10 μm-thick longitudinal cryosections were cut using a Microm HM5000 cryostat and stained with hematoxylin-eosin (HE) to locate the transplantation site. Further immunohistochemical analysis were done using either a mouse anti-human mitochondrial antigen (hMA) monoclonal antibody (Chemicon, MAB1273, 1/50 dilution) for human cell identification, a goat anti-EGFP polyclonal antibody (Abcam, AB6673, 1/1200 dilution) to detect EGFP transgene expression by hMSC cell transplants, and a mouse anti-rat CD68 monocyte/macrophage monoclonal antibody (Chemicon, MAB1435, 1/150 dilution) to detect macrophage infiltration into the transplantation site. In brief, slides were rinsed with a commercial washing buffer (DAKO S3006) and endogeneous peroxidase sites were blocked following 30 min incubation with methanol + 1% hydrogen peroxide. Next, slides were washed with water and washing buffer, followed by incubation with normal serum for 1 hour at room temperature (species dependent on the secondary antibody; normal goat serum (Dako, X0907) for staining with MAB1435 and normal rabbit serum (Dako, X0902) for staining with AB6673 and MAB1273). Subsequently slides were incubated overnight at 4°C with the primary antibody. Following this, slides were rinsed with washing buffer and incubated for 1 hour at room temperature either with peroxidase-coupled rabbit anti-goat Ig antibody (Rockland, 605-4302, 1/200 dilution) to detect EGFP, with peroxidase-coupled goat anti-mouse Ig antibody (Rockland, 610-1319, 1/200 dilution) to detect CD68, or with biotin-coupled rabbit anti-mouse Ig (Abcam, ab67271, 1/200 dilution) to detect hMA. For the latter, an extra incubation step with a HRP-coupled streptavidin based detection complex (StreptABComplex, Dako) was required and performed as described in the manufacturers' instructions. Visualisation for all slides was carried out after staining with DAB (Diaminobenzidine, Dako), according to manufacturers instructions, and nuclei were counterstained with hematoxylin carazzi. Fluorescence imaging (standard 2000 ms exposure time) and bright-field immuno-histochemical analysis was done using an Olympus Bx41 microscope equipped with an Olympus DP50 camera. Olympus DP Software was used for image collection and Photoshop for image processing.

## Authors' contributions

MWR carried out cell culture, cell transplantations, histological analysis, data collection, data interpretation and drafted the manuscript. JD carried out plasmid DNA construction, cell culture and molecular analysis. GS carried out laser capture micro-dissection experiments. SC assisted in planning and evaluation of histological analysis. KV assisted in planning and evaluation of real-time PCR analysis. PD'H assisted in planning and evaluation of laser capture micro-dissection experiments. VFIVT acquired funding and assisted in evaluation of cell culture experiments. EVM assisted in evaluation of histological analysis. DY assisted in evaluation of animal experiments. ZNB acquired funding and assisted in study design and evaluation of cell culture experiments. PGJ acquired funding and assisted in study design and evaluation of animal experiments. PP carried out study design, data collection, data interpretation and drafted the manuscript. All authors have read and approved the final manuscript.
